# Evaluation of Vitamin D3 and Calcium Deficiency after Recovery from Extensive Burn

**DOI:** 10.29252/wjps.10.1.60

**Published:** 2021-01

**Authors:** Ali Akbar Mohammadi, Asma Shafaeipour

**Affiliations:** 1 Department of Surgery, Shiraz University of Medical Sciences, Shiraz, Iran

**Keywords:** Burn, Calcium, Vitamin D, Parathormone (PTH), Iran

## Abstract

**BACKGROUND:**

Previous studies in pediatric populations have demonstrated that vitamin D deficiency is common in patients with large burns. The aim of the current comparative study was to investigate the serum level of vitamin D in patients with large burns [>20% total body surface area (TBSA)] after 6 months of therapy.

**METHODS:**

This case control study was conducted during 6-month period from 2017 to 2018 in Amiralmomenin Hospital, Shiraz, Iran. Forty two patients with large burns (>20% TBSA) and at least 6 months’ post-burn period were enrolled. Also, 42 healthy and age and sex matched controls from those referring for routine check-ups were included for comparison. None of the patients and controls received vitamin D supplements. The serum level of calcium (Ca), parathormone (PTH) and vitamin D were compared between the groups.

**RESULTS:**

There was no significant difference between the two study groups regarding the baseline characteristics including the age (*p=*0.085), gender (*p=*0.275) and duration of sun exposure (*p=*0.894). We found that those with major burns had significantly higher serum levels of PTH (50.48±26.49 *vs.* 33.64±15.80; *p=*0.001). In addition, the serum levels of vitamin D were significantly lower in burn patients compared to healthy controls (18.15±9.18 *vs.* 31.43±16.27; *p<*0.001).

**CONCLUSION:**

Major burns (≥20% TBSA) are associated with increased serum levels of PTH and decreased serum levels of vitamin D. However, serum levels of calcium are not affected by major burns.

## INTRODUCTION

Vitamin D is a mainstay of many physiological processes in the human body, which makes it a vital mineral in many tissues. The fact that several human tissues have the vitamin D receptor, demonstrates that it has an important role in physiological processes. It has been previously well-established that healthy skin and sun exposure play important roles in maintaining appropriate levels of vitamin D.^1^ Vitamin D plays an important role in bone mineralization and intestinal absorption of calcium (Ca), magnesium (Mg), and phosphorus (P).^1^ Other effects of vitamin D has been reported to be prevention of insulin-dependent diabetes mellitus (DM), malignancies and cardiovascular disease, such as hypertension.^2^

Vitamin D deficiency is associated with osteomalacia, rickets and osteoporosis; which are significant morbidities leading to other disorders, such as pathologic vertebral fractures, osteoarthritis and malignancies.^3^ It has been demonstrated that vitamin D deficiency and insufficiently are among the most common public health problems among normal population leading to several morbidities.^4^ The prevalence of the vitamin D deficiency varies widely among different ethnical and geographical groups, which makes it hard to interpret and calculate.^5^ Overall, it has been reported that the rate of vitamin D deficiency reaches 76% in critically ill patients,^6^ while in normal population, it has been estimated to be 81.3%.^4^

Although the clinical importance of the vitamin D deficiency has been studied in several severe diseases and disorders, but the role of vitamin D metabolism and deficiency in burn patients has not been well studied and the effects of vitamin D deficiency on the outcome of patients with large burns remains a dilemma to the physicians.^7^^,^^8^ A limited number of studies have investigated the role of vitamin D in metabolism and outcome of patients with large burns.^6^^, ^^9^^-^^12^ After large burns, the effective surface area of the skin is significantly reduced, leading to a decreased effective surface for vitamin D synthesis. In addition, the dressing and skin coverage for long period of time for treatment of these large burns leads to decreased sun exposure and in turn decreased vitamin D synthesis.^12^

This vicious cycle leads to decreased vitamin D metabolism and production and thus reduced intestinal absorption of Ca, Mg and P, which causes secondary consequences such as decreased bone mineralization and increased risk of other chronic disorders.^6^ In addition, the decreased levels of vitamin D leads to decreased healing rate of the large burns, which aggravates the vicious cycle.^12^ Previous studies in pediatric populations, have demonstrated that vitamin D deficiency is common in patients with large burns and the serum level of vitamin D is correlated with the outcome and mortality.^6^^,^^13^^,^^14^

A literature review revealed that vitamin D deficiency is associated with itching, muscle weakness, and neuropathy in patients with large burns.^12^ However, there is scarce of standard comparative studies investigating the rate and role of vitamin D deficiency in patients suffering from large burns. Thus, the aim of the current comparative study was to investigate the serum level of vitamin D in patients with large burns [>20% total body surface area (TBSA)] after 6 months of therapy when compared to healthy individuals.

## MATERIALS AND METHODS

This case control study was conducted during a 6-month period from December 2017 to June 2018 in Amiralmomenin Hospital, a tertiary health care and referral center of burn and plastic surgery affiliated with Shiraz University of Medical Sciences, Shiraz, Iran. We included all patients with large burns (>20% TBSA) referring to our center during the study period with at least 6 months’ post-burn period. Only thermal burns were included. Those with burns <20% TBSA, those with less than 6 months of follow-up and those who had injuries other than burn; such as fractures and traumatic brain injury (TBI) were excluded from the study. 

Also, those with previous burns, skin cancers, and those who had previously undergone skin surgeries with any etiology were excluded. Those taking vitamin D supplements within the previous 6 months of the study were not included too. Those with severe obesity (BMI>30 kg/m^2^), gastrointestinal surgeries, those suffering from inflammatory bowel disease (IBD) and malabsorption syndromes; such as celiac disease, gastrointestinal malignancies, metabolic disorders and those with endocrinopathy were also excluded from the study. 

A group of individuals referring to the clinic as visitors or for routine check-ups were included as controls. Among them, those with history of burns and skin diseases were not included. Regarding the controls, those with severe obesity (BMI>30 kg/m^2^), gastrointestinal surgeries, those suffering from inflammatory bowel disease (IBD) and malabsorption syndromes; such as celiac disease, gastrointestinal malignancies, metabolic disorders and those with endocrinopathy were excluded. None of the cases and controls received vitamin D supplements within the previous 6 months. The cases and controls were matched regarding the age and gender. The study protocol was approved by the institutional review board (IRB) based on **Declaration** of **Helsinki** and Medical Ethics Committee of Shiraz University of Medical Sciences, Shiraz, Iran (n=14343-01-01-1396). All cases and the controls provided their informed written consents before inclusion in the study. 

All patients were visited in an outpatient clinic by the attending physician and the general surgery resident. The data were extracted from the medical records of the patients. We used a data gathering form to extract and record the data. We recorded the demographic information; such as age and gender, the clinical characteristics of the burn injury including the burn percentage, burn etiology, the duration of the burn, comorbidities, and the outcome. The treatments received during the initials admission were also recorded. The same information was also recorded form the healthy controls, who were eligible to be included in the study. From all patients and the controls, 5 mL of venous blood was withdrawn and was sent to the Amiralmomenin Laboratory for further analysis according to the similar instructions and by the same team. 

Serum level of 25(OH)D, PTH and calcium was measured in all patients and the controls. Immunochemiluminometric assay was performed on a DiaSorin LIAISON (Stillwater, MN) instrument. This highly automated test measured 25-OH-D automatically. The serum level of PTH was quantified by electrochemiluminescence immunoassay (ECLIA). Serum calcium level was measured using the enzyme-linked immunosorbent assay (ELISA) according to the manufacture instructions. Laboratory values were categorized to be normal as follows: 25(OH)D, 25-74 ng/mL; Ca: 8.6-10.2 mg/dL and PTH, 15-65 pg/mL. The inter- and intra-observer reliability was 0.7; thus, there was no heterogeneity among the reported results. 

Based on 80% power to detect at least 5% difference between the primary endpoint of the study, which was the serum level of 25(OH)D with an α equal to 0.05, at least 40 patients in each study group was required. In order to compensate for non-evaluable patients, a total number of 42 patients were included in each study group encompassing 84 total cases and controls. The Statistical Package for Social Science, SPSS for Windows (SPSS Inc., version 22.0, Chicago, Ill., USA) was used for data analysis. All data were presented as mean±SD and proportions were appropriate. The proportions were compared using the Chi-Square test. The parametric variables with normal distribution were compared using the independent t-test. The parametric variables without normal distribution were compared using the Mann-Whitney U test. A 2-sided p-value of less than 0.05 was considered statistically significant.

## RESULTS

Overall, 42 patients with major burns (>20% TBSA) who had at least 6 months of follow-up were included and 42 healthy controls who were matched by age and gender were used for comparison. As demonstrated in Table 1, there was no significant difference between the two study groups regarding the baseline characteristics. The mean age of the patients was 33.60±10.32 years and the mean age of the controls was 30.21±7.14 years. There was no significant difference between the two study groups regarding the age (*p=*0.085). Overall, the mean sun exposure was 2.17±2.24 hours per day among those with burn, before the injury. In the same way, the mean sun exposure was reported to be 2.24±2.23 hours per day in controls. There was no significant difference between the two study groups regarding the mean duration of sun exposure before the injury (*p=*0.894).

**Table 1:** Baseline characteristics of 42 patients with major burns and 42 healthy controls being included in the current study

**Variable**	**Burn patients (n=42)**	**Controls (n=42)**	***p*** ** value**
Age (years)	33.60±10.32	30.21±7.14	0.085
Gender			
Men (%)	23 (45.8)	25 (59.5)	0.275
Women (%)	19 (45.2)	17 (40.5)	
BMI (kg/m^2^)	24.86±8.61	23.74±7.43	0.254
Sun Exposure (hours)	2.17±2.24	2.24±2.23	0.894
Duration of Burn (months)	7.04±1.08	⎼	⎼
TBSA (%)	36.26±12.74	⎼	⎼
Comorbidities (%)	2 (4.8)	1 (2.4)	0.485
Hypertension (%)	1 (2.4)	0 (0.0)	
Hyperthyroidism (%)	1 (2.4)	0 (0.0)	


Table 2 summarizes the results of laboratory analysis of vitamin D, calcium and PTH between the patients and controls. We found that the serum levels of calcium were comparable between the two study groups and there was no significant difference between the two study groups regarding the serum levels of calcium (*p=*0.780). In the same way, the distribution of calcium level based on the identified cut-off values was comparable between the two study groups (*p=*0.070), as demonstrated in Table 2. The serum level of PTH was significantly higher (*p=*0*.*001) in patients with major burns, when compared to healthy controls. However, there was no significant difference between the two study groups (*p=*0.502) regarding the distribution of the PTH using the proposed cut-off values. The serum level of vitamin D was significantly lower (*p<*0.001) in patients with major burns, when compared to healthy controls. However, there was no significant difference between the two study groups (*p=*0.502) regarding the distribution of the PTH using the proposed cut-off values. 

**Table 2:** The serum levels of calcium, parathormone and vitamin D in burn patients compared to healthy controls

**Variable**	**Burn patients (n=42)**	**Control (n=42)**	***p*** ** value**
Calcium (mg/dL)	9.69±0.65	9.66±0.28	0.780
Normal (%)	37 (88.1)	42 (100.0)	0.070
Deficient (%)	1 (2.4)	0 (0.0)	
High (%)	4 (9.5)	0 (0.0)	
PTH (pg/mL)	50.48±26.49	33.64±15.80	0.001
Normal (%)	33 (78.5)	37 (88.1)	0.502
Deficient (%)	2 (4.8)	1 (2.4)	
High (%)	7 (16.7)	4 (9.5)	
Vitamin D (ng/mL)	18.15±9.18	31.43±16.27	<0.001
Normal (%)	9 (21.4)	28 (66.6)	<0.001
Deficient (%)	33 (78.6)	13 (31.0)	
High (%)	0 (0.0)	1 (2.4)	


PTH: Parathormone

## DISCUSSION

Vitamin D plays a crucial role in normal physiology and metabolism of human body and its deficiency is associated with severe morbidities.^1^ Major burns are associated with loss of effective body surface for synthesis and production of human required vitamin D. This results in vitamin D deficiency, which decreases the intestinal absorption of calcium and other metabolites and thus retards the skin regeneration process. This leads to formation of a vicious cycle which finally is associated with severe vitamin D deficiency and impaired skin regeneration and healing. In the current study, the serum levels of calcium, PTH and vitamin D were investigated in patients with major burn injuries. 

We found that the serum levels of calcium were comparable between cases and controls, 6 months after the injury. However, major burns were associated with increased levels of PTH and decreased levels of vitamin D, 6 months after the injury. This means that major burns result in decreased serum levels of vitamin D, which in turn leads to increased serum levels of PTH to compensate the decreased levels of calcium. The effect of vitamin D deficiency on outcome and complications of patients with burns has been investigated addressing the children and pediatric population. One study demonstrated that 62.3% of the burn children have low serum levels of calcidiol or calcitriol during the acute phase of the burn and intensive care unit (ICU) admission.^6^ Gottschlich *et al.* administered ergocalciferol for 2 weeks at 800 IU daily for children younger than 3 years and 1600 IU daily for those older. They increased the dosage each week by double to reach the maximum dosage of 4,000 IU daily. They reported that those receiving the vitamin D supplementation had finally lower serum levels of calcidiol and calcitriol when compared to those who did not received supplements of vitamin D. They concluded that malabsorption after burn has resulted in decreased serum level of vitamin D even after supplementation.^6^

In another study, Kelin *et al.* administered 400 IU of vitamin D daily to pediatric patients with large burns. They demonstrated that the administered dosage of vitamin D failed to correct the deficiency appropriately. This resulted in the fact that in burn patients, oral supplements cannot correct the serum level of vitamin D.^15^ Both studies suffered from a shortcoming which was the choice of supplement used. It is well-established that ergocalciferol is less effective than cholecalciferol on raising serum levels of vitamin D, especially in burn patients.^1^ In a recent study, Gottschlich *et al.* compared the efficacy of vitamin D2 and D3 in critically ill burn patients. They demonstrated that all patients suffered from severe vitamin D deficiency one year after burn injuries. Based on their results, they recommended continued treatment with vitamin D3 beyond the acute phase post-burn to counteract the trajectory of abnormal serum levels and associated morbidity.^10^

Currently, the literature is scarce of isolated studies on adult burn patients and serum level of vitamin D and its effects on the outcome of patients. However, it has been demonstrated that burn patients suffer from decreased serum levels of calcidiol. The pathophysiology of vitamin D deficiency in patients with burn is a vicious cycle. The burn results in decreased surface area of the skin, which is the mainstay of the vitamin D deficiency. In addition, the prolonged hospitalization and use of dressing for a long-term resulted in decreased sun exposure and decreased vitamin D synthesis leading to vitamin D deficiency. In return, the vitamin D deficiency resulted in delayed wound healing and skin regeneration, which aggravated the vicious cycles and resulted in more decline in serum level of vitamin D.^9^^,^^12^

In burn patients, the effective surface for production of vitamin D is decreased. In addition, the biosynthetic functions of skin are severely impaired after burn injury. This results in decreased levels of 7-dehydrocholesterol in the remnant skin, and thus its subsequent conversion into cholecalciferol following exposure to UV light is significantly decreased.^16^ In addition, abnormalities and impairment of the calcium-parathyroid hormone (Ca-PTH) axis after the thermal injury leads to aggravated cycle of the vitamin D synthesis leading to decreased levels of vitamin D. The pro-inflammatory state induced by burn injury leads to upregulation of PTH calcium receptor, which in turn leads to inappropriately low levels of circulating PTH and decreased action of 1-α hydroxylase in the kidney. This results in a deficiency of metabolically active vitamin D.^11^

Adams and Hewison performed a review to evaluate the changes in metabolism and pathway of vitamin D in burn patients. They demonstrated that replenishment of vitamin D levels can be considered in two phases of restoration and maintenance. Restoration of vitamin D levels requires larger doses to be given than for maintenance, because the body stores have a large volume of distribution. However, recent research suggests that giving large doses of vitamin D may itself cause adverse effects.^17^ Treatment of vitamin D deficiency may also improve common symptoms suffered by burn patients, such as pruritis. A retrospective case series of patients with idiopathic pruritis, rashes, and angioedema treated with vitamin D led to complete resolution of symptoms in a significant proportion of the cohort.^18^ This has the potential to improve patient quality of life, in addition to the prevention of deficiency-related complications.

Further investigation is required regarding the prevalence of vitamin D deficiency in adult burn patients. Although numerous studies focus on the effect of vitamin D deficiency on bone quality in post-burn injury patients,^9^^,^^12^^,^^13^ there are little data available on the correlation between decreased vitamin D levels and the common post-burn sequelae of itch, muscle weakness, and peripheral neuropathy. Symptom relief in the acute care setting also requires investigation as possible outcome measures for deficiency. Further studies are also needed to determine the most appropriate form of supplementation, adequate dosing regimens, and potential need for long-term supplementation.

We noted some limitations in our study. First, we included a limited number of patients with major burns and age- and sex-matched controls. This might have results in under-power bias in the findings. However, we have run a post-analysis power calculation and found that the study had at least 80% power to detect at least 5% difference between the serum level of vitamin D. Further studies with larger sample population and probably multicenter ones are required. Second, we did not determine the outcome measures. Thus, we cannot comment on the effects of vitamin D deficiency and the outcome and complications of the burn injuries. In other words, the clinical significance of the vitamin D deficiency in these patients should be identified and investigated in the future studies. The other limitation of the current study was that we only measured the serum level of 25(OH)D and no other metabolites of the vitamin D. Thus, we cannot comment on the etiology and pathomechanism of the vitamin D deficiency in patients with major burns. Taking all together, it is among the only available controlled comparative studies, addressing the rate of the vitamin D deficiency in patients with major burns. 

## CONCLUSION

Major burns (≥20% TBSA) are associated with increased serum levels of PTH and decreased serum levels of vitamin D. However, serum levels of calcium are not affected by major burns. Based on the results of the current study, we recommend addition of vitamin D supplements in patients with major burns. Further studies are required to investigate the clinical effects of vitamin D deficiency in patients with major burns.
